# Cognitive decline and quality of life in incident Parkinson's disease: The role of attention

**DOI:** 10.1016/j.parkreldis.2016.04.009

**Published:** 2016-06

**Authors:** Rachael A. Lawson, Alison J. Yarnall, Gordon W. Duncan, David P. Breen, Tien K. Khoo, Caroline H. Williams-Gray, Roger A. Barker, Daniel Collerton, John-Paul Taylor, David J. Burn, ICICLE-PD study group

**Affiliations:** a Institute of Neuroscience, Newcastle University, Newcastle upon Tyne, UK; b Centre for Clinical Brain Sciences, University of Edinburgh, Edinburgh, UK; c John van Geest Centre for Brain Repair, University of Cambridge, UK; d School of Medicine & Menzies Health Institute Queensland, Griffith University, Australia

**Keywords:** Parkinson's disease, Quality of life, Mild cognitive impairment, Dementia, Attention

## Abstract

**Introduction:**

Parkinson's disease dementia (PDD) is associated with poorer quality of life (QoL). Prior to the onset of PDD, many patients experience progressive cognitive impairment. There is a paucity of longitudinal studies investigating the effects of cognitive decline on QoL. This study aimed to determine the longitudinal impact of cognitive change on QoL in an incident PD cohort.

**Methods:**

Recently diagnosed patients with PD (n = 212) completed a schedule of neuropsychological assessments and QoL measures; these were repeated after 18 (n = 190) and 36 months (n = 158). Mild cognitive impairment (PD-MCI) was classified with reference to the Movement Disorder Society criteria. Principal component analysis was used to reduce 10 neuropsychological tests to three cognitive factors: attention, memory/executive function, and global cognition.

**Results:**

Baseline PD-MCI was a significant contributor to QoL (β = 0.2, p < 0.01). For those subjects (9%) who developed dementia, cognitive function had a much greater impact on QoL (β = 10.3, p < 0.05). Multivariate modelling showed attentional deficits had the strongest predictive power (β = −2.3, p < 0.01); brief global tests only modestly predicted decline in QoL (β = −0.4, p < 0.01).

**Conclusions:**

PD-MCI was associated with poorer QoL over three years follow up. Cognitive impairment had a greater impact on QoL in individuals who developed dementia over follow-up. Impaired attention was a significant determinant of QoL in PD. Interventions which improve concentration and attention in those with PD could potentially improve QoL.

## Highlights

• Rate of QoL decline was three times faster in PD-MCI subjects compared those with normal cognition.
• Cognitive decline predicted worsening of QoL over 36 months.
• Decline in attention was the strongest cognitive predictor of declining QoL.

## 1 Introduction

Cognitive impairment is a common non-motor symptom in Parkinson's disease (PD), with up to 80% of patients eventually developing dementia (PDD) [1]. PDD is distressing for patients and their carers and has been significantly associated with poor quality of life (QoL) in people with PDD and carer burden [2]. As PD progresses, motor and non-motor symptoms, can negatively impact on QoL and wellbeing [3], [4], but a large prospective study found the development of non-motor symptoms over two years had the greatest impact on QoL [4].

It is vital to understand which factors may impact on QoL in those with PD. It has been suggested that cognitive impairment may impair activities of daily living (ADL) [5], resulting in poorer QoL. Cognitive impairment has been shown to reduce task-oriented coping in PD patients which contributed to poorer QoL [6]. However, there is a paucity of studies investigating the long term changes of QoL in PD, and in particular, the association with changes in cognitive function.

This study investigated the longitudinal relationship between cognitive function and QoL from PD diagnosis to 36 month follow up. A secondary aim was to determine whether there was an optimal measure of cognition in our schedule of neuropsychological assessments which would predict QoL. We hypothesised that participants who demonstrated greater cognitive decline over 36 months would have a significantly poorer QoL compared with subjects who maintained a stable or normal cognition during the follow-up period.

## 2 Methods

### 2.1 Participants

**Fig. 1:**
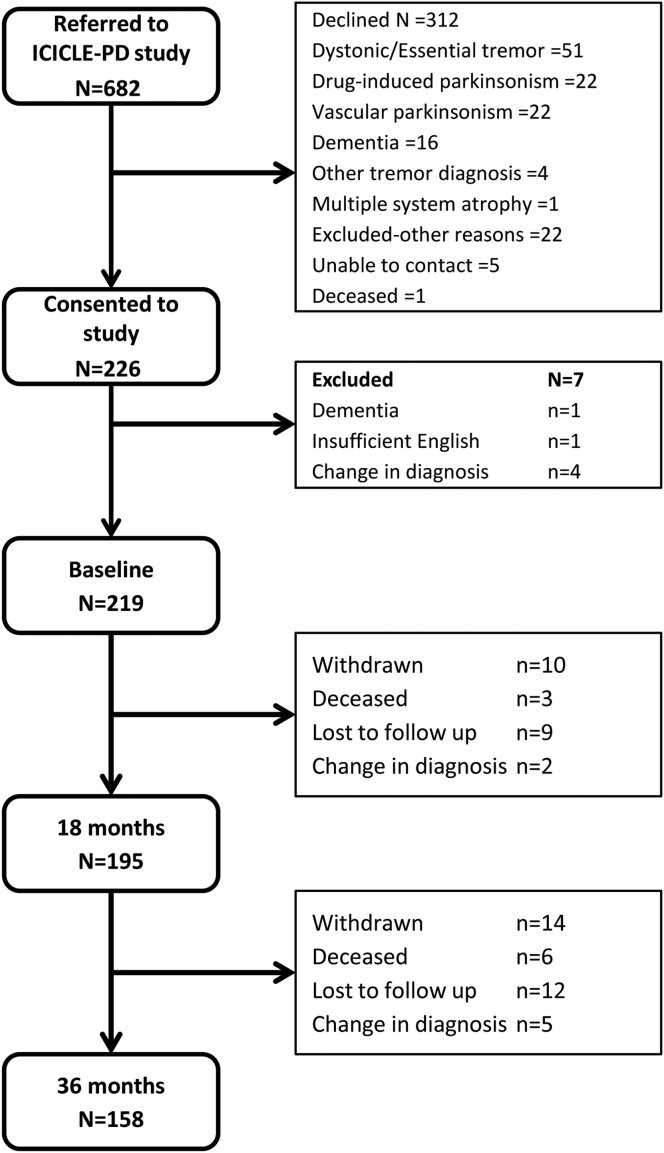
Flow diagram of subject participation. ICICLE-PD study = Incidence of cognitive impairments in cohorts with longitudinal evaluation in Parkinson's disease.

Between June 2009 and December 2011, newly diagnosed PD patients from community and outpatient clinics in Newcastle-upon-Tyne, Gateshead and Cambridgeshire, UK were invited to participate in the Incidence of Cognitive Impairments in Cohorts with Longitudinal Evaluation in Parkinson's disease (ICICLE-PD) study [7]. Participants were subsequently re-assessed at 18 month intervals. Idiopathic PD was diagnosed by a movement disorder specialist and fulfilled Queen's Square Brain Bank criteria [8]. Of 682 people invited to take part in the ICICLE-PD study, 226 consented, 312 declined to take part and the remaining individuals were excluded (Fig. 1). Participants were excluded if they had significant cognitive impairment at presentation (Mini Mental State Examination (MMSE) < 24) or a pre-existing diagnosis of dementia [9]. Healthy control subjects (n = 99) were recruited from the community to provide age, sex and culturally appropriate normative data. Power calculations determined 98% power (effect size = 0.05) for PD vs. control groups (α = 0.05, two tailed).

This study was approved by the Newcastle and North Tyneside Research Ethics Committee. All subjects provided written informed consent at each time point. Capacity was assessed in accordance with the Mental Capacity Act 2005, England; participants were deemed to have capacity to consent if they were able to satisfy the researcher that they understood they were attending for a research study and relate back to the researcher the tests and assessments they would be asked to do. All participants in this study had the capacity to give informed consent.

### 2.2 Assessments

Demographic information, including age, sex, and education was collected. Participants completed the Movement Disorder Society (MDS) Unified Parkinson's Disease Rating Scale (MDS-UPDRS) Part III [10], the National Adult Reading Test (NART) [11] as a measure of pre-morbid IQ, and the Geriatric Depression Scale (GDS-15) [12]. Participants were assessed in an “on” motor state. Levodopa equivalent dose (LED) was calculated using methods described by Tomlinson et al. [13]. The summary index of the Parkinson's Disease Questionnaire (PDQ-39) [14] was used to measure global QoL and is well validated; scores ranged from 0 (best possible QoL) to 100 (worst possible QoL).

Participants completed a schedule of neuropsychological tests. Global cognitive function was assessed using the MMSE [15] and Montreal Cognitive Assessment (MoCA) [16]. Attention was measured using Power of Attention (PoA) and Digit Vigilance Accuracy (percentage of correct targets detected) from the Cognitive Drug Research (CDR) battery [17]. PoA is a composite score of the mean time (msec) for Simple Reaction Time, Choice Reaction Time and Digit Vigilance reaction time. Memory was assessed using the number of correct answers from Pattern Recognition Memory and Spatial Recognition Memory, and mean trials to success for Paired Associate Learning from the Cambridge Neuropsychological Test Automated Battery (CANTAB) [18]. Executive function was assessed using the One Touch Stockings of Cambridge from CANTAB (number correct on first trial), phonemic fluency (number of word generated in 60s beginning with the letter F) and semantic fluency (number of animals generated in 90s). Visuospatial function was evaluated using the pentagon copying item within the MMSE and was graded using a modified 0–2 rating scale [19]. Language was assessed using the naming (0–3) and sentence (0–2) items in the MoCA.

Our schedule of neuropsychological tests preceded the introduction of the MDS criteria for mild cognitive impairment (PD-MCI) [20]; therefore, we used a modified MDS Level II criteria, described previously by Yarnall et al. [7]. Briefly, participants were classified as PD-MCI if they performed two standard deviations (SD) or more below the mean of appropriate norms (controls) on at least two neuropsychological tests across five cognitive domains: attention, memory, executive function, language and visuospatial function. For data that were not normally distributed, percentiles derived from a normal distribution were used to estimate cut-offs [7]. A 2 SD cut off was used as recent studies suggest this is the optimal cut off to distinguish PD-MCI from normal cognition (PD-CN) [21]. Subjective cognitive decline and functional independence were determined through semi-structured interviews with participants and/or their carers to enable PDD diagnosis using the MDS criteria in appropriate cases [9].

### 2.3 Statistical analysis

Statistical analyses were performed using SPSS software (Version 21.0; SPSS, Inc., Chicago, IL). Data were examined for normality of distribution with visual histograms and Kolmogorov-Smirnov's test. Comparisons of means between two groups were performed using independent t-tests or Mann-Whitney U tests, depending on distribution. For more than two group comparisons, one way ANOVA or Kruskal-Wallis tests were used as appropriate. Repeated measures were examined using repeated measures ANOVA or Friedman tests. Spearman's rank correlation was used to test associations between variables. Multiple comparisons underwent Bonferroni's correction. Principal component analysis (PCA) using oblique oblimin rotation was used to reduce the large number of highly correlated neuropsychological assessments to a smaller number of independent cognitive dimensions. This allowed an evaluation of whether certain domains or groups of tests could be used to predict QoL. Hierarchical regression was used to determine significant predictors of QoL. Backwards stepwise regression was used to determine a basic model of predictors involving: age, sex, years of education, LED, GDS-15 score and MDS-UPDRS III; non-significant predictors were excluded. Measures of cognition were then entered to produce a final model.

R software (Version 3.0.1; R Foundation for Statistical Computing, Vienna, Austria) and *lme4* were used to perform linear mixed effects analysis of the relationship between QoL and cognition from baseline to 36 months. A random intercept model was used, where the intercept varied at the participant and time level. Age, sex, years of education, LED, GDS-15 score and MDS-UPDRS III score were entered into the model as fixed effects, as well as interactions of time with depression score (GDS-15 × Time), motor severity (Time × MDS-UPDRS III) and LED (Time × LED). A reduced model was produced by excluding non-significant predictors to which cognitive measures were added. Fit of the models was assessed by likelihood ratio tests.

## 3 Results

At baseline, 219 participants completed assessments; over 36 months, seven participants were re-diagnosed as not having idiopathic PD (Fig. 1) and were excluded, leaving 212 PD participants. In addition, 24 participants withdrew from the study, 21 were not contactable (lost to follow up), and nine participants died. Thus 158 of 212 participants (75%) returned for evaluation at 36 months (mean time interval 3.1 ± 0.2 years). Seven participants at 18 months and nine participants at 36 months did not complete the PDQ-39 questionnaire and were excluded from the cross-sectional analysis. There were no significant differences in baseline demographics, global cognition or QoL between completers and those lost to further evaluation (Supplementary Table 1).

At baseline, 21% of participants were classified as PD-MCI at 2 SDs below normative values. By 36 months, 14 participants (9%) had developed PDD and 27% were classified as PD-MCI; seven participants (4%) with baseline PD-MCI had reverted to normal cognition. Participants with cognitive impairment were significantly older, had completed fewer years of education and had lower pre-morbid IQ (Table 1, p < 0.05 for all at each time point). At 18 and 36 month evaluation, post hoc analysis revealed that PD-MCI and PDD participants had more severe motor disease than those with normal cognition (PD-CN) (p < 0.01).

PDQ-39 scores were significantly higher in those with PD-MCI compared to PD-CN at baseline (24.4 ± 16.5 vs. 16.8 ± 13.2, respectively, p < 0.01), 18 months (26.8 ± 18.9 vs. 17.9 ± 14.3, respectively, p < 0.01), and 36 months (25.0 ± 16.8 vs. 18.2 ± 14.9, respectively, p < 0.01). QoL was significantly poorer in patients with PDD compared to those with PD-MCI at 36 months (40.1 ± 17.9 vs. 25.0 ± 16.8, respectively, p < 0.01).

### 3.1 Principal component analysis

Principal component analysis using baseline data identified three principal components: attention, memory/executive function and global cognition (Supplementary Table 2). These principal components accounted for 62% of the variance of baseline cognition (12%, 40% and 10% respectively, for each component). Factor scores were then calculated using the component score coefficient matrix at baseline, 18 months and 36 months. Lower scores indicated poorer cognitive function in each test.

Correlation analysis indicated a weak but significant association between PDQ-39 and the attention factor score at baseline (ρ = −0.21, p < 0.01) after Bonferroni corrections were applied. At 18 months, weak but significant correlations were observed between QoL and memory/executive function (ρ = −0.27, p < 0.01) and global cognition (ρ = −0.29, p < 0.01); attention was also significantly correlated but not after Bonferroni corrections were applied (ρ = −0.20, p < 0.05). At 36 months, the correlation of PDQ-39 scores with attention (ρ = −0.42, p < 0.01), memory/executive function (ρ = −0.50, p < 0.01) and global cognition (ρ = −0.39, p < 0.01) were stronger, suggesting an increasing association over 36 months.

### 3.2 Baseline predictors of quality of life

Repeated measures analysis showed PDQ-39 scores significantly increased over time in participants with PD-MCI at baseline (24.4 ± 16.5 vs. 27.9 ± 18.2 vs. 32.7 ± 20.8 at baseline, 18 and 36 months, respectively, χ^2^ = 11.2, p < 0.01), with a mean paired change of 9.1 ± 15.0. This was compared to PD-CN participants at baseline with a mean paired change of 3.0 ± 13.3 in PDQ-39 scores over 36 months (χ^2^ = 7.6, p < 0.05).

Hierarchical regression was used to determine baseline predictors of QoL at 36 months. Backwards stepwise regression was used to determine that fewer years in education, higher baseline motor severity and higher baseline depression scores were significant predictors of poorer QoL 36 months later (p < 0.05 for all). This accounted for 30% of the variance (adjusted R^2^ = 0.30, F = 21.9, p < 0.01).

Baseline MoCA score as a measure of global cognition, cognitive classification (PD-CN or PD-MCI), and factor scores from the PCA analysis were separately added to the basic model. Small but significant changes were observed by adding baseline MoCA scores and cognitive classification, accounting for 3% (ΔR^2^ = 0.03, p < 0.05) and 4% (ΔR^2^ = 0.04, p < 0.01) respectively of the total variance (Table 2). In contrast, baseline factor scores of cognition resulted in the largest percentage change, accounting for 13% of the variance of QoL at 36 months (ΔR^2^ = 0.13, p < 0.01). However, only baseline attention was significant (p < 0.01). The regression model including baseline attention alone explained 11% of the variance (ΔR^2^ = 0.11, p < 0.01).

### 3.3 Longitudinal analysis of cognition and quality of life

Linear mixed effects modelling was used to determine the association between changing cognition and QoL using all 212 participants. Significant predictors of longitudinal PDQ-39 scores are shown in Table 3; being younger, female, fewer years of education, higher LED and persistent depression predicted poorer QoL over 36 months. Increasing PD motor severity over time (β = 0.2, p < 0.01), but not cross-sectional disease motor severity (β = 0.1, p > 0.05), predicted decreasing QoL. The model also suggested that time, in itself, was associated with an improvement in QoL (β = −3.5, p < 0.01).

MoCA, cognitive classification and PCA factor scores plus interactions with time were separately added to the basic model (Table 3). Decreasing MoCA scores over time predicted decline in QoL. Neither PD-MCI classification nor PDD at a particular time point were significant predictors of QoL. However, the interaction of PDD with time was a significant predictor of decreasing QoL scores (β = 10.3, p < 0.05), suggesting that developing PDD over time significantly reduces an individual's QoL. The PCA factor scores did not significantly predict cross-sectional QoL. However, declining attention over time was a significant predictor of decreasing QoL (β = −2.3, p < 0.001).

Log-likelihood ratio comparing the fit of the models showed the basic model plus PCA factor scores was a significantly stronger model compared to the basic model, MoCA or cognitive classification (χ^2^ = 578.8, p < 0.001). Therefore, changes in attention may be more accurate in predicting poorer long term QoL.

## 4 Discussion

This is the first study to investigate the longitudinal effects of cognitive impairment on QoL using a range of validated assessments in a large group of patients with newly diagnosed PD. We have demonstrated that cognitive impairment contributes to longitudinal QoL change in people with PD, and that decline in attention has the greatest predictive power.

Participants with PD-MCI at baseline reported a mean increase of nine points in PDQ-39 scores over 36 months, indicating that QoL deteriorated over time. This magnitude of change has been suggested to be clinically significant [22] and was three times greater compared to participants classified as PD-CN at baseline, which would not be regarded as clinically meaningful change.

Baseline PD-MCI was a predictor of poorer QoL at 36 months, although the proportion of variance explained was small. Another longitudinal study reported similar findings, albeit with a shorter duration of follow-up and less comprehensive neuropsychological assessments [3]. In our study, it was the development of dementia, rather than PD-MCI, that was associated with decline in QoL. This is consistent with a cross-sectional study by Leroi et al. [2] who reported that subjects with PDD had significantly worse QoL than participants with normal cognition or PD-MCI.

A small but significant change in QoL at 36 months was predicted by lower MoCA scores at diagnosis. Multilevel modelling showed declining MoCA scores over 36 months predicted decreasing QoL. Therefore, the MoCA could be potentially useful to clinicians; it is a measure of global cognition that is quick and simple to administer, but could have utility in anticipating future difficulties for individuals with PD.

However, greater predictive accuracy may come from the use of more specific cognitive assessments. PCA permitted the evaluation of clusters of cognitive tests in relation to QoL. Attention was the only significant predictor of QoL, either as a baseline predictor or using multilevel modelling. Baseline attention factor scores also accounted for the largest change in variance between models of PDQ-39 scores at 36 months, and a stronger predictive power compared with MoCA or cognitive classification longitudinally. This is notable, since few studies have evaluated specific cognitive domains with regards to QoL. Klepak et al. [23] observed that poorer visual attention/memory was associated with poorer QoL, whilst another study also found that participants with attentional/memory deficits reported increased PDQ-39 scores [24]. However, neither of these studies examined attention as an isolated cognitive domain. One study found that attentional deficits in PDD were significantly detrimental to both basic and instrumental ADL [5]. These include activities such as: washing, dressing, eating and managing medications, as well as social interactions, keeping appointments and engagement in leisure activities, which are important in maintaining good quality of life. Impaired attention is a feature of PDD [25]; therefore, attention may be an alternative marker for the development of PDD and it could be postulated that it is actually incipient PDD that is more important for QoL. Nonetheless, our findings have implications for clinicians as interventions targeting attention could significantly improve QoL.

Younger age, higher depression scores and increased disease severity were associated with poorer QoL, both in cross-sectional and longitudinal analysis. This is consistent with previous studies [4], [23], [26]. Being female predicted having poorer QoL, as did increased LED score. Gender as a predictor of QoL is an inconsistent finding in the literature with better QoL being reported in males, females or no significant differences at all [23], [26]. Years in education seemed to be a protective factor, which could be indicative of the role of education and IQ in cognitive reserve [27]. Interestingly, increasing time as a variable predicted improved QoL scores. This could indicate a degree of successful adjustment and coping in the absence of declining motor symptoms and cognition, which is consistent with findings in other studies [6].

This study pre-dated the MDS PD-MCI criteria and is therefore limited in its assessment of language and visuospatial function, therefore a modified version was used. However, the concept of PD-MCI has been debated and as yet PD-MCI criteria have not been validated, with cut offs of 1, 1.5 and 2 SD below normative values all being used. 312 patients declined participation, which may introduce selection bias. As with many longitudinal studies, missing data were problematic (Supplementary Table 3). This reduced the statistical power that requires complete datasets, such as linear regression and PCA. The missing CDR data could have affected PD-MCI classification at 36 months with the false-negative (Type I error) classification of PD-CN in some participants. However, differences between participants were examined graphically and analytically; participants were representative of the whole sample, with no significant differences in global cognition or QoL scores. Additionally, linear mixed effect modelling is able to handle missing data and does not remove participant data listwise. The small number of participants who did not return for further evaluation may have been those with a more rapid decline in PD, cognition and QoL and would therefore have been of particular interest to this study. However, baseline scores tested analytically and graphically did not reveal any significant differences. Some participants improved in their neuropsychological assessment scores which could be due to a learning effect, medication effects or normal fluctuations in cognition. We used a time interval of 18 months between testing, which has been suggested as an appropriate length of time to negate practice effects. Finally, we used the PDQ-39 as a PD specific QoL measure; it has been suggested that a generic should also be used when using disease-specific QoL measures. However, the PDQ-39 is well validated and recommended by some for measuring QoL in PD [28].

In summary, cognitive impairment had a significant, albeit small, role in determining QoL. Over three years following diagnosis, most patients were cognitively stable and for them QoL was not greatly influenced by cognition. Patients who were categorised as having PD-MCI had worse QoL scores. A minority of people developed PDD over three years, where cognition had a much greater impact on QoL. Brief clinical tests could predict to some degree those people at risk of declining QoL, but more sophisticated attentional tasks may have greater predictive power. Pharmacological interventions, in the form of rivastigmine [29], and non-pharmacological interventions, such as cognitive rehabilitation focused on attention [30], may be useful in improving attention and concentration in PD patients and, therefore, QoL. Further longitudinal studies are required to substantiate the findings of this study and to better evaluate the impact of treatments for attentional decline.

## Author roles

RA Lawson was involved with coordination of the study, participant recruitment, data collection, statistical analysis, interpretation of data and drafted the manuscript.

AJ Yarnall, GW Duncan and DP Breen were also involved with coordination of the study, participant recruitment, clinical assessment, data collection and manuscript revision.

TK Khoo was involved with the study design and coordination of the study. He was also involved with participant recruitment, clinical assessment, data collection and manuscript revision.

CH Williams-Gray was involved with study co-ordination, clinical assessment, data collection and manuscript revision.

RA Barker is a principal investigator and co-applicant for the main funding grant. He was involved with the study design and reviewed the manuscript.

D Collerton and JP Taylor were involved with data interpretation and manuscript revision.

DJ Burn is the chief investigator and main applicant for the funding grant. He was involved with the study design, supervised the study, data interpretation and reviewed the manuscript.

## Funding

ICICLE-PD was funded by Parkinson's UK (J-0802) and Lockhart Parkinson's Disease Research Fund. The research was supported by the National Institute for Health Research (NIHR) Newcastle Biomedical Research Unit based at Newcastle upon Tyne Hospitals NHS Foundation Trust and Newcastle University and a NIHR Biomedical Research Centre award to the University of Cambridge/Addenbrooke's Hospital.

RA Lawson is supported by grants from the Lockhart Parkinson's Disease Research Fund. AJ Yarnall is funded by the Biomedical Research Unit, Newcastle University, and has previously been supported by grants from the Lockhart Parkinson's Disease Research Fund and the Michael J. Fox Foundation (MJFF). She has received honoraria from Teva-Lundbeck and sponsorship from Teva-Lundbeck, UCB, GlaxoSmithKline (GSK), Genus, Britannia Pharmaceuticals Ltd. and AbbVie for attending conferences. GW Duncan has no financial disclosures. DP Breen has received speaker fees from UCB and Britannia Pharmaceuticals Ltd. TK Khoo has no financial disclosures. CH Williams-Gray has received honoraria from Lundbeck, an educational grant from UCB Pharma, faculty stipends and travel expenses from the Movement Disorder Society, and receives grant support from the Academy of Medical Sciences, the Rosetrees Trust, Stevenage Biosciences Catalyst and Addenbrooke's Charitable Trust. RA Barker receives editorial monies from Springer and royalties from Wiley. He has grant support from NIHR; Parkinson's UK; Cure Parkinson's Trust; Rosetrees Trust; MRC; ACT and EU. D Collerton has no financial disclosures. JP Taylor has been a consultant for Lundbeck, GE Healthcare and Novartis. He has received speaker fees from GE Healthcare and Flynn pharmaceuticals. DJ Burn has received grants from NIHR, Wellcome Trust, and Parkinson's UK. He has received speaker fees from Acadia Pharmaceuticals.

## Conflict of interest

None.

## Figures and Tables

**Table 1:** Demographic and clinical characteristics of participants by cognitive classification at baseline, 18 months and 36 months.

	Baseline (n = 212)			18 months (n = 183)				36 months (n = 149)			
PD-CN (n = 167)	PD-MCI (n = 45)	p Value	PD-CN (n = 124)	PD-MCI (n = 51)	PDD (n = 8)	p Value	PD-CN (n = 95)	PD-MCI (n = 40)	PDD (n = 14)	p Value
Age (years)	65.1 (9.9)	68.7 (9.0)	0.024^a^	66.1 (9.4)	72.3 (8.3)	74.8 (6.3)	<0.001^b^	66.5 (9.2)	71.9 (8.7)	75.3 (7.4)	<0.001 ^b^
Gender (male)^†^	104 (62)	30 (67)	0.728^c^	77 (62)	33 (65)	5 (63)	0.948^c^	59 (62)	28 (70)	10 (71)	0.566^c^
Education (years)	13.2 (3.5)	11.3 (3.5)	<0.001^d^	13.4 (3.5)	11.2 (2.9)	12.1 (4.4)	<0.001^e^	13.5 (3.5)	11.9 (2.9)	12.1 (3.9)	0.003^e^
NART	115.9 (9.4)	108.7 (11.4)	<0.001^d^	116.8 (9.6)	110.3 (10.0)	107.3 (11.7)	<0.001^e^	117.3 (8.0)	109.4 (12.6)	107.3 (11.0)	<0.001^e^
UPDRS III Total	26.5 (11.0)	31.2 (14.2)	0.064^d^	30.1 (11.1)	40.7 (12.0)	48.1 (8.9)	<0.001^e^	31.9 (12.9)	41.1 (15.0)	46.5 (15.8)	<0.001^e^
Hoehn and Yahr stage	1.9 (0.6)	2.1 (0.8)	0.039^d^	2.1 (0.5)	2.4 (0.6)	2.6 (0.7)	0.004^e^	2.0 (0.5)	2.2 (0.6)	2.7 (0.8)	<0.001^e^
LED (mg/d)	175.7 (160.4)	190.1 (133.8)	0.188^d^	420.7 (233.0)	430.5 (256.1)	303.1 (123.6)	0.329^e^	512.4 (289.2)	588.4 (297.5)	493.1 (239.9)	0.585^e^
GDS-15	2.6 (2.4)	3.8 (3.2)	0.013^d^	2.5 (2.5)	3.5 (3.2)	3.0 (1.3)	0.074^e^	2.7 (2.4)	3.5 (2.6)	4.5 (2.8)	0.018^e^
PDQ-39	16.8 (13.2)	24.4 (16.5)	0.004^d^	17.9 (14.3)	26.6 (18.9)	28.3 (13.9)	0.006^e^	18.2 (14.9)	25.0 (16.8)	40.1 (17.9)	<0.001^e^
MoCA^‡^	26.2 (2.7)	22.3 (4.0)	<0.001^d^	27.3 (2.7)	24.4 (3.1)	17.4 (3.7)	<0.001^e^	27.3 (2.4)	23.8 (3.2)	19.6 (4.3)	<0.001^e^
MMSE	28.9 (1.1)	28.0 (1.6)	<0.001^d^	28.9 (1.3)	27.6 (1.7)	25.0 (2.2)	<0.001^e^	28.9 (1.4)	27.4 (2.4)	24.4 (3.4)	<0.001^e^

All figures are mean(standard deviation) except † where figures are n(%), ‡ at baseline n = 23 did not complete MoCA.

a = independent *t*-test, b = ANOVA, c = Chi squared test, d = Mann-Whitney *U* test, e = Kruskal-Wallis.

PD-CN= Parkinson's disease with normal cognition, PD-MCI = Mild cognitive impairment in Parkinson's disease using the 2 standard deviation cut-off, PDD = Parkinson's disease dementia, NART = National Adult Reading Test, UPDRS III = Movement Disorders Society-Unified Parkinson's Disease Rating Scale Part III, LED = Levodopa equivalent dose, GDS-15 = Geriatric Depression Scale, PDQ-39 = Parkinson's Disease Questionnaire, MoCA = Montreal Cognitive Assessment, MMSE = Mini-Mental State Examination.

**Table 2:** Regression coefficients and model fit of baseline cognitive predictors of quality of life at 36 months.

Predictors in model^a^	β	t	p	95% CI for β		R	R^2^	Adj R2	Std. Error	Δ R^2^
Lower bound	Upper bound
Baseline MoCA	−0.17	−2.27	0.025	−1.6	−0.11	0.58	0.33	0.31	14.11	0.03
Baseline PD-MCI	0.2	2.91	0.004	2.86	14.95	0.6	0.35	0.34	13.7	0.04
Baseline factor scores	–	–	–	–	–	0.64	0.41	0.38	13.36	0.13
Baseline Memory/Executive function factor score	−0.11	−1.28	0.203	−5.4	1.16	–	–	–	–	–
Baseline Attention factor score	−0.3	3.64	<0.001	2.27	7.68	–	–	–	–	–
Baseline Global cognition factor score	−0.09	−1.03	0.305	−4.8	1.52	–	–	–	–	–
Baseline Attention factor score	−0.35	4.48	<0.001	3.26	8.42	0.63	0.39	0.37	13.5	0.11

MoCA = Montreal Cognitive Assessment, PD-MCI 2 SD = PD-MCI classified using the Movement Disorder Society with 2 standard deviation (SD) cut off.

a Model also includes: years of education, Movement Disorders Society-Unified Parkinson's Disease Rating Scale Part III score and Geriatric Depression Scale score.

**Table 3:** Predictors of quality of life using mixed effects modelling.

	β	Std. Error	t	p	
**Basic model**					
Gender (Female)	3.7	1.3	2.7	0.007	**
Education (Years)	−0.7	0.2	−3.6	<0.001	***
Age	−0.3	0.1	−4.4	<0.001	***
LED	0.02	0.0	3.8	<0.001	***
GDS-15	3.3	0.4	8.2	<0.001	***
Time (Assessment)	−3.5	1.3	−2.6	0.009	**
UPDRS III	0.1	0.1	0.3	0.785	
UPDRS III over Time	0.2	0.0	5.1	<0.001	***
GDS-15 over Time	−1.1	0.1	−8.2	<0.001	***
**Basic model** + **MoCA**					
MoCA	0.2	0.3	0.8	0.448	
MoCA over Time	−0.4	0.1	−3.1	0.002	**
**Basic model** + **Cognitive classification**					
PD-MCI	2.1	2.6	0.8	0.433	
PDD	−16.6	10.9	−1.5	0.129	
PD-MCI over Time	0	1.2	0	0.973	
PDD over Time	10.3	4.0	2.6	0.011	*
**Basic model** + **Factor scores**					
Memory/executive function	1.0	1.3	0.7	0.457	
Attention	1.9	1.3	1.5	0.13	
Global Cognition	0.1	1.3	0.0	0.971	
Memory/executive function over Time	−1.1	0.6	−1.6	0.107	
Attention over Time	−2.3	0.7	−3.5	<0.001	***
Global Cognition over Time	−0.4	0.6	−0.7	0.492	

* p < 0.05, ** p < 0.01, *** p < 0.001.

GDS-15 = Geriatric Depression Scale, UPDRS III = Movement Disorders Society-Unified Parkinson's Disease Rating Scale Part III, LED = Levodopa equivalent dose, MoCA = Montreal Cognitive Assessment, PD-MCI = PD-MCI classified using the Movement Disorder Society with 2 standard deviation (SD) cut off, PDD = Parkinson's disease dementia.

## References

[1] Hely M.A., Reid W.G., Adena M.A., Halliday G.M., Morris J.G. (2008). The Sydney multicenter study of Parkinson's disease: the inevitability of dementia at 20 years. Mov. Disord..

[2] Leroi I., McDonald K., Pantula H., Harbishettar V. (2012). Cognitive impairment in Parkinson disease: impact on quality of life, disability, and caregiver burden. J. Geriatr. Psychiatry Neurol..

[3] Visser M., Verbaan D., van Rooden S., Marinus J., van Hilten J., Stiggelbout A. (2009). A longitudinal evaluation of health-related quality of life of patients with Parkinson's disease. Value Health.

[4] Antonini A., Barone P., Marconi R., Morgante L., Zappulla S., Pontieri F., Ramat S., Ceravolo M., Meco G., Cicarelli G., Pederzoli M., Manfredi M., Ceravolo R., Mucchiut M., Volpe G., Abbruzzese G., Bottacchi E., Bartolomei L., Ciacci G., Cannas A., Randisi M., Petrone A., Baratti M., Toni V., Cossu G., Dotto P., Bentivoglio A., Abrignani M., Scala R., Pennisi F., Quatrale R., Gaglio R., Nicoletti A., Perini M., Avarello T., Pisani A., Scaglioni A., Martinelli P., Iemolo F., Ferigo L., Simone P., Soliveri P., Troianiello B., Consoli D., Mauro A., Lopiano L., Nastasi G., Colosimo C. (2012). The progression of non-motor symptoms in Parkinson's disease and their contribution to motor disability and quality of life. J. Neurol..

[5] Bronnick K., Ehrt U., Emre M., De Deyn P.P., Wesnes K., Tekin S., Aarsland D. (2006). Attentional deficits affect activities of daily living in dementia-associated with Parkinson's disease. J. Neurol. Neurosurg. Psychiatry.

[6] Hurt C.S., Landau S., Burn D.J., Hindle J.V., Samuel M., Wilson K., Brown R.G. (2012). Cognition, coping, and outcome in Parkinson's disease. Int. Psychogeriatr..

[7] Yarnall A.J., Breen D.P., Duncan G.W., Khoo T.K., Coleman S.Y., Firbank M.J., Nombela C., Winder-Rhodes S., Evans J.R., Rowe J.B., Mollenhauer B., Kruse N., Hudson G., Chinnery P.F., O'Brien J.T., Robbins T.W., Wesnes K., Brooks D.J., Barker R.A., Burn D.J. (2013). Characterizing mild cognitive impairment in incident Parkinson disease: the ICICLE-PD Study. Neurology.

[8] Hughes A.J., Daniel S.E., Kilford L., Lees A.J. (1992). Accuracy of clinical diagnosis of idiopathic Parkinson's disease: a clinico-pathological study of 100 cases. J. Neurol. Neurosurg. Psychiatry.

[9] Emre M., Aarsland D., Brown R., Burn D.J., Duyckaerts C., Mizuno Y., Broe G.A., Cummings J., Dickson D.W., Gauthier S., Goldman J., Goetz C., Korczyn A., Lees A., Levy R., Litvan I., McKeith I., Olanow W., Poewe W., Quinn N., Sampaio C., Tolosa E., Dubois B. (2007). Clinical diagnostic criteria for dementia associated with Parkinson's disease. Mov. Disord..

[10] Goetz C.G., Tilley B.C., Shaftman S.R., Stebbins G.T., Fahn S., Martinez-Martin P., Poewe W., Sampaio C., Stern M.B., Dodel R., Dubois B., Holloway R., Jankovic J., Kulisevsky J., Lang A.E., Lees A., Leurgans S., LeWitt P.A., Nyenhuis D., Olanow C.W., Rascol O., Schrag A., Teresi J.A., van Hilten J.J., LaPelle N. (2008). Movement disorder society-sponsored revision of the unified Parkinson's disease rating scale (MDS-UPDRS): scale presentation and clinimetric testing results. Mov. Disord..

[11] Mathias J.L., Bowden S.C., Barrett-Woodbridge M. (2007). Accuracy of the Wechsler test of Adult reading (WTAR) and national Adult reading test (NART) when estimating IQ in a healthy Australian sample. Aust. Psychol..

[12] Yesavage J.A., Brink T.L., Rose T.L., Lum O., Huang V., Adey M., Leirer V.O. (1982). Development and validation of a geriatric depression screening scale: a preliminary report. J. Psychiatr. Res..

[13] Tomlinson C.L., Stowe R., Patel S., Rick C., Gray R., Clarke C.E. (2010). Systematic review of levodopa dose equivalency reporting in Parkinson's disease. Mov. Disord..

[14] Jenkinson C., Fitzpatrick R.A.Y., Peto V.I.V., Greenhall R., Hyman N. (1997). The Parkinson's Disease Questionnaire (PDQ-39): development and validation of a Parkinson's disease summary index score. Age Ageing.

[15] Folstein M.F., Folstein S.E., McHugh P.R. (1975). Mini-mental state”. A practical method for grading the cognitive state of patients for the clinician. J. Psychiatr. Res..

[16] Nasreddine Z.S., Phillips N.A., Bédirian V., Charbonneau S., Whitehead V., Collin I., Cummings J.L., Chertkow H. (2005). The montreal cognitive assessment, MoCA: a brief screening tool for mild cognitive impairment. J. Am. Geriatr. Soc..

[17] Nicholl C.G., Lynch S., Kelly C.A., White L., Simpson P.M., Wesnes K.A., Pitt B.M.N. (1995). The cognitive drug research computerized assessment system in the evaluation of early dementia-is speed of the essence?. Int. J. Geriatr. Psychiatry.

[18] Robbins T.W., James M., Owen A.M., Sahakian B.J., McInnes L., Rabbitt P. (1994). Cambridge Neuropsychological Test Automated Battery (CANTAB): a factor analytic study of a large sample of normal elderly volunteers. Dementia.

[19] Ala T.A., Hughes L.F., Kyrouac G.A., Ghobrial M.W., Elble R.J. (2001). Pentagon copying is more impaired in dementia with Lewy bodies than in Alzheimer's disease. J. Neurol. Neurosurg. Psychiatry.

[20] Litvan I., Goldman J.G., Tröster A.I., Schmand B.A., Weintraub D., Petersen R.C., Mollenhauer B., Adler C.H., Marder K., Williams-Gray C.H., Aarsland D., Kulisevsky J., Rodriguez-Oroz M.C., Burn D.J., Barker R.A., Emre M. (2012). Diagnostic criteria for mild cognitive impairment in Parkinson's disease: movement Disorder Society Task Force guidelines. Mov. Disord..

[21] Goldman J.G., Holden S., Bernard B., Ouyang B., Goetz C.G., Stebbins G.T. (2013). Defining optimal cutoff scores for cognitive impairment using movement disorder society task force criteria for mild cognitive impairment in Parkinson's disease. Mov. Disord..

[22] Clarke C.E., Furmston A., Morgan E., Patel S., Sackley C., Walker M., Bryan S., Wheatley K. (2009). Pilot randomised controlled trial of occupational therapy to optimise independence in Parkinson's disease: the PD OT trial. J. Neurol. Neurosurg. Psychiatry.

[23] Klepac N., Trkulja V., Relja M., Babic T. (2008). Is quality of life in non-demented Parkinson's disease patients related to cognitive performance? A clinic-based cross-sectional study. Eur. J. Neurol..

[24] Barone P., Antonini A., Colosimo C., Marconi R., Morgante L., Avarello T.P., Bottacchi E., Cannas A., Ceravolo G., Ceravolo R., Cicarelli G., Gaglio R.M., Giglia R.M., Iemolo F., Manfredi M., Meco G., Nicoletti A., Pederzoli M., Petrone A., Pisani A., Pontieri F.E., Quatrale R., Ramat S., Scala R., Volpe G., Zappulla S., Bentivoglio A.R., Stocchi F., Trianni G., Dotto P.D. (2009). The PRIAMO study: a multicenter assessment of nonmotor symptoms and their impact on quality of life in Parkinson's disease. Mov. Disord..

[25] Ballard C.G., Aarsland D., McKeith I., O'Brien J., Gray A., Cormack F., Burn D., Cassidy T., Starfeldt R., Larsen J.P., Brown R., Tovee M. (2002). Fluctuations in attention - PD dementia vs DLB with parkinsonism. Neurology.

[26] Behari M., Srivastava A.K., Pandey R.M. (2005). Quality of life in patients with Parkinson's disease. Park. Relat. Disord..

[27] Armstrong M.J., Naglie G., Duff-Canning S., Meaney C., Gill D., Eslinger P.J., Zadikoff C., Mapstone M., Chou K.L., Persad C., Litvan I., Mast B.T., Fox S., Tang-Wai D.F., Marras C. (2012). Roles of education and IQ in cognitive reserve in Parkinson's disease-mild cognitive impairment. Dement. Geriatr. Cogn. Dis. Extra.

[28] Martinez-Martin P., Jeukens-Visser M., Lyons K.E., Rodriguez-Blazquez C., Selai C., Siderowf A., Welsh M., Poewe W., Rascol O., Sampaio C., Stebbins G.T., Goetz C.G., Schrag A. (2011). Health-related quality-of-life scales in Parkinson's disease: critique and recommendations. Mov. Disord..

[29] Mamikonyan E., Xie S.X., Melvin E., Weintraub D. (2015). Rivastigmine for mild cognitive impairment in Parkinson disease: a placebo-controlled study. Mov. Disord..

[30] Cerasa A., Gioia M., Salsone M., Donzuso G., Chiriaco C., Realmuto S., Nicoletti A., Bellavia G., Banco A., D’amelio M., Zappia M., Quattrone A. (2014). Neurofunctional correlates of attention rehabilitation in Parkinson's disease: an explorative study. Neurol. Sci..

