# Supplementary material for: Cognitive decline and quality of life in incident Parkinson's disease: The role of attention
Source: Parkinsonism Relat Disord. 2016 Jun;27:47–53. doi: 10.1016/j.parkreldis.2016.04.009 (PMC4906150; doi:10.1016/j.parkreldis.2016.04.009)
Supplement: Supplementary file 1 [file mmc1.docx]

Supplementary Table 1: Comparison of baseline demographic and clinical characteristics of participants who returned for 36 month evaluation (completers) and non-completers of assessments

|  | Completers  (n=158) | | Non-completers  (n=54) | | t/z | p |
| --- | --- | --- | --- | --- | --- | --- |
|  | Mean | SD | Mean | SD |  |  |
| *Age (years)* | 65.5 | 9.4 | 66.8 | 10.9 | -0.8 | 0.47 |
| *Education (years)* | 12.9 | 3.4 | 12.6 | 4.1 | -0.9 | 0.54 |
| *NART* | 114.3 | 10.5 | 114.8 | 9.8 | -0.4 | 0.81 |
| *UPDRS III Total* | 26.6 | 10.9 | 30.2 | 14.3 | -1.5 | 0.20 |
| *Hoehn and Yahr stage* | 1.9 | 0.7 | 2.0 | 0.7 | -0.5 | 0.57 |
| *LED (mg/d)* | 172.3 | 140.0 | 197.7 | 192.6 | -0.1 | 0.87 |
| *GDS-15* | 2.7 | 2.5 | 3.3 | 2.8 | -1.6 | 0.18 |
| *PDQ-39* | 18.3 | 13.8 | 18.8 | 15.8 | -0.2 | 0.93 |
| *MoCA^†^* | 25.4 | 3.5 | 25.2 | 3.3 | -0.6 | 0.51 |
| *MMSE* | 28.8 | 1.2 | 28.7 | 1.5 | -0.1 | 0.96 |
|  | n | % | n | % | χ^2^ | p |
| *Gender (male)* | 103 | 65 | 31 | 57 | 0.4 | 0.19 |

NART = National Adult Reading Test, UPDRS III = Movement Disorders Society-Unified Parkinson’s Disease Rating Scale Part III, LED = Levodopa equivalent dose, MoCA = Montreal Cognitive Assessment, MMSE = Mini Mental State Examination, GDS-15 = Geriatric Depression Scale, NPI = Neuropsychiatric Inventory, PDQ-39 = Parkinson’s Disease Questionnaire.

† For MoCA, Completers n= 139, Non-completers n= 49.
