# Supplementary material for: Cognitive decline and quality of life in incident Parkinson's disease: The role of attention
Source: Parkinsonism Relat Disord. 2016 Jun;27:47–53. doi: 10.1016/j.parkreldis.2016.04.009 (PMC4906150; doi:10.1016/j.parkreldis.2016.04.009)
Supplement: Supplementary file 2 [file mmc2.docx]

Supplementary Table 2: Factor loadings based on principal component analysis with oblimin rotation

|  |  | Component |  |
| --- | --- | --- | --- |
|  | Memory/ Executive function factor | Attention factor | Global cognition factor |
| *PAL* | -0.81 |  |  |
| *PRM* | 0.78 |  |  |
| *SRM* | 0.72 |  |  |
| *OTS* | 0.70 |  |  |
| *PoA* |  | 0.87 |  |
| *Digit Vigilance* |  | -0.81 |  |
| *Verbal fluency* |  |  | 0.83 |
| *Semantic fluency* |  |  | 0.55 |
| *MMSE* |  |  | 0.64 |
| *MoCA* |  |  | 0.63 |

MoCA = Montreal Cognitive Assessment, MMSE = Mini Mental State Examination, PoA = Power of attention, PRM = Paired Recognition Memory, SRM = Spatial Recognition Memory, PAL = Paired Associated Learning, OTS = One Touch Stockings.
