# Supplementary material for: Cognitive decline and quality of life in incident Parkinson's disease: The role of attention
Source: Parkinsonism Relat Disord. 2016 Jun;27:47–53. doi: 10.1016/j.parkreldis.2016.04.009 (PMC4906150; doi:10.1016/j.parkreldis.2016.04.009)
Supplement: Supplementary file 3 [file mmc3.docx]

Supplementary Table 3: Missing data

|  | Baseline (n=212) | | 18 months (n=190) | | 36 months (n=158) | |
| --- | --- | --- | --- | --- | --- | --- |
|  | Number missing | Reason for missing data | Number missing | Reason for missing data | Number missing | Reason for missing data |
| *PDQ-39* | - | - | 7 | Missing data | 9 | Missing data |
| *MoCA* | 24 | Introduced later in study | 1 | Missing data | 6 | Missing data |
| *CDR* | 2 | Equipment failure | 3 | Missing data | 32 | Data collection problems |
| *CANTAB* | 11 | Visual impairment (n=3),  missing data (n=8) | 4 | Visual impairment (n=2),  missing data (n=1),  equipment failure (n=1) | 11 | Visual impairment (n=1),  missing data (n=10) |
| *Verbal fluency* | 2 | Missing data | 1 | Missing data | 3 | Missing data |
| *Semantic fluency* | 3 | Missing data | 2 | Missing data | 4 | Missing data |

PDQ-39 = Parkinson’s Disease Questionnaire, MoCA = Montreal Cognitive Assessment, CDR = Cognitive Drug Research, CANTAB = Cambridge Neuropsychological Test Automated Battery.
